# Is traumatic stress research global? A bibliometric analysis

**DOI:** 10.3402/ejpt.v5.23269

**Published:** 2014-02-20

**Authors:** Kinga E. Fodor, Johanna Unterhitzenberger, Chia-Ying Chou, Dzenana Kartal, Sarah Leistner, Maja Milosavljevic, Agnes Nocon, Laia Soler, Jenifer White, Seonyoung Yoo, Eva Alisic

**Affiliations:** 1 Department of Clinical Psychology, Faculty of Medicine, Semmelweis University, Budapest, Hungary; 2 Department of Clinical and Biological Psychology, Catholic University Eichstaett-Ingolstadt, Eichstätt, Germany; 3 Department of Clinical, Educational and Health Psychology, University College London, London, United Kingdom; 4 Department of Psychology and Psychiatry, Monash University, Melbourne, Australia; 5 Department of Psychiatry, Australian Centre for Posttraumatic Mental Health, University of Melbourne, Melbourne, Australia; 6 Institute of Mental Health, Belgrade, Serbia; 7 Department of Personality, Assessment and Psychological Treatment, University of Barcelona, Barcelona, Spain; 8 International Psychology Department, The Chicago School of Professional Psychology, Chicago, IL, USA; 9 Graduate School of Comprehensive Human Sciences, University of Tsukuba, Japan; 10 Monash Injury Research Institute, Monash University, Melbourne, Australia; 11 National Psychotrauma Center for Children and Youth, University Medical Center Utrecht, Utrecht, The Netherlands

**Keywords:** Bibliometric analysis, systematic review, capacity building, global mental health, low- and middle-income countries, posttraumatic stress disorder, traumatic stress research

## Abstract

**Background:**

The representation of low- and middle-income countries (LMIC) in traumatic stress research is important to establish a global evidence base, build research capacity, and reduce the burden of unmet mental health needs around the world. Reviews of the traumatic stress literature up to 2002 showed trends toward globalization although LMIC were only marginally represented compared to high-income countries (HIC).

**Objective:**

To examine the global nature of current traumatic stress research. In particular, we were interested in the extent to which traumatic stress research is: (1) conducted in LMIC, (2) conducted by LMIC researchers, and (3) accessible to them.

**Method:**

Using the databases PubMed, PsychInfo, and PILOTS, we systematically searched for peer-reviewed articles on traumatic stress published in any language in the year 2012. Out of the 3,123 unique papers identified, we coded a random sample (*N*=1,000) for study, author, article, and journal characteristics.

**Results:**

Although our sample involved research in 56 different countries, most papers (87%) involved research in HIC, with 51% of all papers describing studies in the United States. In 88% of the papers, the author team was affiliated with HIC only. Less than 5% of all author teams involved collaborations between HIC and LMIC researchers. Moreover, 45% of the articles on LMIC studies published by a HIC corresponding author did not involve any LMIC co-authors. LMIC researchers appeared to publish empirical studies in lower impact journals. Of the 1,000 articles in our sample, 32% were open access and 10% were made available via different means; over half of the papers were not accessible without subscription.

**Conclusions:**

Traumatic stress research is increasingly global but still strongly dominated by HIC. Important opportunities to build capacity in LMIC appear to be missed. Implications toward more international traumatic stress research are discussed.

Traumatic experiences range from collective events like mass violence, war, terrorism, and natural disasters to personal, even “everyday life” traumas such as road traffic accidents and the sudden loss of a loved one. People around the world are affected by such experiences and the aftermath of trauma is an international matter. Nine years ago, Bedard, Greif, and Buckley (2004) suggested that with an increasing awareness of violence occurring across the globe, there is a greater need for traumatic stress research stemming from all cultures and societies. In a similar vein, Schnyder (2013) stated that: (1) trauma is a global issue; (2) traumatic stress research needs worldwide, interdisciplinary collaborations over competition; and (3) the traumatic stress research community needs to ensure that all trauma related research and mental health needs are met regardless of nationality.

Nevertheless, there is evidence that traumatic stress research has not been evenly occurring in different areas of the world (Bedard et al., 2004; Figueira et al., 2007; Olff & Vermetten, 2013; Patel & Sumathipala, 2001). For example, in a bibliometric review of the posttraumatic stress disorder (PTSD) literature between 1983 and 2002, Figueira et al. (2007) reported that overall, 69% of the papers originated from the United States. This percentage decreased from 88% in the period 1983–1987 to 62% in the period 1998–2002. Although the number of publishing countries increased (36 countries contributed to PTSD literature in total), only 25% (*n*=9) counted as low- and middle-income countries (LMIC) according to the current classification by The World Bank (2012).

Today, 83% of the world's population live in LMIC, with the fastest growth of population occurring in the countries with the lowest incomes (United Nations, Department of Economic and Social Affairs, Population Division, 2013). The risk of experiencing a potentially traumatic event and developing mental health disorders has been reported to be higher in countries with a low economic status (Demyttenaere et al., 2004) due to the risk factors associated with poverty, social exclusion (Patel, 2001; Patel & Kleinman, 2003) and experiences of loss, trauma, and displacement (e.g., De Jong et al., 2001; Fazel, Wheeler, & Danesh, 2005; Steel et al., 2009). Often, the beneficial effects of research do not extend to these regions, leading to inequalities (e.g., Saxena et al., 2011). LMIC face a significant burden of unmet mental health needs including trauma-related challenges (World Health Organization, 2001). To reduce this strain and narrow the gap between high-income countries (HIC) and LMIC, a comprehensive knowledge base is needed. Well-designed policies that lead to cost effective, evidence-based, feasible interventions are essential to effective health care practice and can only be derived from research (Patel, 2000; Sharan et al., 2007). Therefore, to achieve adequate mental health care systems around the world, research into posttraumatic mental health should be just as global as the impact of the phenomenon.

To design strategies to promote a comprehensive, global evidence base on traumatic stress, it is essential to have a clear picture of the nature of the recent body of literature. Our current insight in the status of traumatic stress research around the world is based on analyses of the literature of more than a decade ago. The trend of internationalization up to 2002 as described by Bedard et al. (2004) and Figueira et al. (2007) may have continued, accelerated or stalled, with potentially important implications for future research and funding efforts.

We aimed to assess the current standing of traumatic stress research in terms of its global nature and to provide insight in factors identified as affecting research gaps between HIC and LMIC (Lansang & Dennis, 2004). These variables, such as the accessibility of research literature and international collaborations, are fundamental necessities for building research capacity. In particular, our goal was to answer the following questions:

1. To what extent are traumatic stress studies *conducted in* LMIC?
2. To what extent are traumatic stress studies *conducted by* researchers from LMIC?
3. To what extent is traumatic stress literature *accessible to* researchers from LMIC?

## Method

This study has been conducted in the context of the “Young Minds Paper in a Day” event at the 2013 conference of the European Society for Traumatic Stress Studies in Bologna. “Paper in a Day” brings young researchers together to stimulate international connections and collaborations (Alisic, 2012). Our team came from Australia, Germany, Hungary, Japan, Serbia, Spain, the UK, and the USA. Many of us did not know each other beforehand. All team members participated in the preparations for the event, the day itself and the subsequent stages of the study, including design, coding, analyses, and manuscript writing.

### Selection of articles

We conducted a systematic search for peer-reviewed papers on traumatic stress, published in 2012. References from PubMed, PsychInfo and PILOTS (the “Published International Literature on Traumatic Stress” database maintained by the US National Center for PTSD) were included. The following MeSh terms and keywords were used for PsychInfo: *posttraumatic stress disorder.SH OR stress reactions.SH OR PTSD.TI OR PTSD.AB OR post traumaticstress.TI OR post traumaticstress.AB OR posttraumatic stress.TI OR posttraumatic stress.AB OR post-traumatic stress.TI OR post-traumatic stress.AB OR traumatic stress.TI OR traumatic stress.AB*, which was subsequently adapted to PubMed requirements. For PILOTS, specific keywords were not required because the entire database is focused on traumatic stress research. We conducted our search on 20 April, 2013 and restricted it to peer-reviewed publications from the year 2012. All languages were included. The search resulted in 1895 peer-reviewed papers in PsychInfo, 1960 in PubMed and 1,353 in PILOTS, of which 2,076 were duplicates. Of the 3,132 unique papers, we took a random sample of *N*=1,000 (via SPSS's random sampling option within the “select cases” function) to be further coded and analyzed.

### Coding and analysis

For each study, bibliographic information was automatically retrieved (title, author, journal title, abstract, journal language). The first round of coding relied on the abstracts and, if necessary, full-text articles. We coded three variables. First, we recorded whether an article reported empirical human research or not. Empirical human research was defined as original research that examined human subjects (e.g., treatment outcome, case, or epidemiological studies). For the empirical studies, we subsequently recorded the country in which the study was conducted. In the few cases in which military personnel was surveyed while on duty, their country of origin was noted. Finally, we recorded the country with which the corresponding author was primarily affiliated. Two authors independently coded 11% of the papers, with Cohen's kappa as a measure of agreement. Kappa was 0.92 (empirical vs. non-empirical paper), 0.98 (study country), and 0.99 (country of corresponding author), respectively.

For the second round of coding, we retrieved the full-text articles. To minimize the loss of data due to different access levels to the journals between our research team members, every study was coded by a pair of team members and all missing values were checked by a third author. We recorded whether an article was open access (and if so official open access or unofficial open access) or not open access (we are aware that some LMIC researchers may have access via subscriptions and some HIC researchers may not be in this position. Based on Sharan et al. (2007), however, we assumed that LMIC researchers in particular have difficulties with access). We also coded the affiliations of the author team (all authors affiliated with HIC, all authors affiliated with LMIC, or author team affiliated with both HIC and LMIC). Third, we noted whether the funding of the work was mentioned and if so, from which country the funding came from. Finally, we recorded whether the article was published in a journal focused on psychotraumatology (e.g., *European Journal of Psychotraumatology*, *Journal of Traumatic Stress*, *Psychological Trauma*) or a broader journal and what the impact factor of the journal was according to the Social Science Citation Index of 2011. For all coding into HIC and LMIC, we used the classification of The World Bank (2012). Every country with less than $12.475 income per capita was classified as a LMIC. For a categorization into continents (a solely geographic categorization), ambiguous areas like Central American states were identified as North American, whereas countries like Russia and Turkey were assigned to Asia. We retrieved percentages of the world population by country from the United Nations’ World Population Prospects paper (United Nations, Department of Economic and Social Affairs, Population Division, 2013).

Analyses were conducted with IBM SPSS Statistics 20.0 and consisted of descriptive statistics (frequencies and proportions), Chi-squares, *t*-tests and ANOVAs. For question 1, we considered the empirical papers (*n=*709) only, because of our focus on whether the world's population was evenly studied in traumatic stress research (e.g., a large number of opinion pieces from a certain area could bias our results). For questions 2 and 3, we decided to consider the total sample of 1,000 papers (only the analysis regarding impact factors revealed a difference between empirical and non-empirical papers, which is described below; the other analyses showed no significant differences).

## Results

Out of the random sample of 1,000 papers, a minority (15.4%; *n=*154) was published in traumatic stress focused journals whereas 84.6% (*n=*846) were found in broader topic journals. Almost all publications were in English (94.6%; *n=*946). Thirteen languages were represented in the non-English articles, with the top three being German (2%; *n=*20), French (0.8%; *n=*8), and Chinese (0.7%; *n=*7). Over two thirds of the articles (70.9%; *n=*709) involved empirical studies on human subjects (further called “empirical”). The other papers (29.1%; *n=*291) included reviews, opinion pieces, theoretical accounts, meta-analyses, and animal studies.

### To what extent are traumatic stress studies conducted in LMIC?

The papers reported on samples from 56 different countries. The vast majority of papers regarded HIC samples (86.5%; *n=*613) whereas 12.7% (*n=*90) regarded LMIC samples and 0.8% (*n=*6) involved both HIC and LMIC. The distribution by continent showed that most papers described studies in North America (55.1%; *n=*391), followed by Europe (23.3%; *n=*166), Asia (12.1%; *n=*88), Australia (3.9%; *n=*28), Africa (2.0%; *n=*15), and South America (1.3%; *n=*9). A further 1.7% (*n=*12) spanned more than one continent. Half of the papers (50.6%; *n=*359) reported on research in the USA, in which 4.5% of the world population live. China, the country with the highest population rate (19.3%), featured in 4.2% (*n=*30) of the articles, whereas India, the second leading country with 17.5% of the world population appeared in 0.1% (*n=*1) of the publications. Table 1 shows all countries and their frequencies with which they featured in empirical research on trauma. Regarding studies involving LMIC for which funding was explicitly mentioned (*n=*46 articles, of which three papers regarded combined LMIC and HIC research), 47.8% (*n=*22) of the papers acknowledged funding by HIC whereas 52.2% (*n=*24) were funded by LMIC.

**Table 1:** Distribution of study countries and primary affiliations of corresponding authors

Country	% of empirical papers on studies conducted in country (*n=*709)	% of corresponding authors’ primary affiliations with country (*n*=1,000)
USA	50.6	53.7
UK	4.8	6.2
Germany	4.8	5.9
Canada	4.2	4.3
China*	4.2	3.1
Australia	3.5	3.0
Netherlands	2.8	2.9
Italy	2.1	2.3
Israel	1.8	1.9
Switzerland	1.4	1.8
France	1.4	1.7
Norway	1.1	1.1
Japan	1.1	1.0
Spain	1.0	0.8
Turkey*	1.0	0.7
Brazil*	0.7	1.2
Poland	0.7	0.8
Denmark	0.7	0.7
South Africa*	0.7	0.5
Belgium	0.6	0.6
Iran*	0.6	0.5
Sweden	0.6	0.5
Palestine*	0.6	0.1
Uganda*	0.6	0.1
Croatia	0.4	0.4
South Korea*	0.4	0.4
Sri Lanka*	0.4	0.1
Pakistan*	0.4	–
New Zealand	0.3	0.4
Hungary	0.3	0.3
United Arab Emirates	0.3	0.3
Portugal	0.3	0.2
Malaysia*	0.3	0.1
Lebanon*	0.3	–
Peru*	0.3	–
Rwanda*	0.3	–
Greece	0.1	0.3
India*	0.1	0.3
Russia*	0.1	0.2
Argentina*	0.1	0.1
Cambodia*	0.1	0.1
DRC*	0.1	0.1
Finland	0.1	0.1
Ireland	0.1	0.1
Jordan*	0.1	0.1
Mexico*	0.1	0.1
Nepal*	0.1	0.1
Scotland	0.1	0.1
Singapore	0.1	0.1
Tanzania*	0.1	0.1
Chile*	0.1	–
Haiti*	0.1	–
Iraq*	0.1	–
Kenya*	0.1	–
Papua New Guinea*	0.1	–
South Sudan*	0.1	–
Egypt*	–	0.2
Bosnia-Herzegovina*	–	0.1
Iceland	–	0.1
Taiwan*	–	0.1

* *Note*: Indicates a country is considered LMIC according to The World Bank (2012).

### To what extent are traumatic stress studies conducted by researchers from LMIC?

In 88.4% (*n=*884) of the cases, all authors were affiliated with a HIC. In 7.1% (*n=*71) of the sample, all authors were affiliated with LMIC and in 4.5% (*n=*45), the team was a combination of HIC and LMIC researchers. Table 2 depicts author affiliation by study sample (HIC, LMIC or combination). Studies conducted in LMIC or in a combination of LMIC and HIC were regularly published by “HIC only” author teams (19.8%; *n=*19 out of 96). The level of concordance between the research teams’ affiliation and the study country differed significantly according to the HIC, LMIC, or mixed nature of the research team (*χ* ^2^(4)=511.31, *p*<0.001).

**Table 2:** Authors’ affiliations regarding research country setting (*n=*709)

	Study country		
	
Authors’ affiliations	HIC (*n=*613)	LMIC (*n=*90)	HIC & LMIC (*n=*6)
Research group			
all HIC	98.7% (*n=*605)	16.7% (*n=*15)	85.7% (*n=*4)
all LMIC	0.8% (*n=*5)	50.0% (*n=*45)	0% (*n*=0)
collaboration HIC and LMIC	0.5% (*n=*3)	33.3% (*n=*30)	14.3% (*n=*2)
			
Corresponding author			
HIC	99.8% (*n=*612)	36.7% (*n=*33)	100% (*n=*6)
LMIC	0.2% (*n=*1)	63.3% (*n=*57)	0% (*n*=0)

HIC=high-income countries; LMIC=low- and middle-income countries.

Due to their status in author teams, we also examined the primary affiliations of corresponding authors. These affiliations involved 50 different countries, led by the United States (53.7%, *n=*537), followed by the United Kingdom (6.2%, *n=*62), Germany (5.9%, *n=*59), and Canada (4.3%, *n=*43). Virtually all corresponding authors (92.0%; *n=*920) were primarily affiliated with HIC. For empirical studies conducted in HIC, 99.8% of corresponding authors’ primary affiliations were HIC, whereas for LMIC studies there was a match in 63.3% of the cases. When HIC researchers published on LMIC studies as corresponding authors (*n*=33), 45% of the articles did not include any LMIC co-authors.

Finally, we considered whether affiliation was related to impact factor of the hosting journal. The mean journal impact factor for articles was 2.84 (SD*=*4.01) for ‘HIC only’ research groups, 2.61 (SD*=*1.54) for HIC-LMIC collaborations, and 1.85 (SD=1.82) for “LMIC only” teams. The differences were non-significant (*F*(2)=2.24, *p=*0.107) despite non-overlapping 95% confidence intervals for HIC and LMIC (note that group sizes were considerably unequal). A direct comparison of the impact factor of publications by “HIC only” versus “LMIC only” teams showed a significant difference (*t*(953)=2.06, *p=*0.040; this finding held for the empirical papers (*t*(672)=2.11, *p*=0.035) separately but not for the non-empirical papers (*t*(279)=1.01, *p*=0.315)). For corresponding authors, HIC affiliation was associated with higher impact factor articles: the mean impact factor was 2.82 (SD*=*3.95) for corresponding authors primarily affiliated with HIC and 2.04 (SD*=*1.73) for those primarily affiliated with LMIC (*t*(164)=3.36, *p*=0.001; equal variances not assumed since Levene's test was significant at 0.046).

### To what extent is traumatic stress literature accessible to researchers from LMIC?

In this section, our main analysis regarded the open-access nature of articles. Of the 1,000 articles, 32.3% (*n=*323) were officially open access, meaning that they were accessible without any subscription. Approximately 10% (*n=*102) of the papers were not officially open access but could nevertheless be retrieved from the internet, for example through author homepages. More than half of the articles were only accessible via subscription (57.5%, *n=*575). There were no significant differences on these three categories of accessibility between papers on studies in HIC and papers on studies in LMIC (*χ* ^2^(4)=4.55, *p*=0.337.

## Discussion

This bibliometric analysis of recent traumatic stress research explored variables that could have meaningful implications for closing the research gap between HIC and LMIC. It revealed that even though there is an increasingly diverse background to recent trauma literature, it is still dominated by HIC and opportunities to build capacity in LMIC are underutilized. In our sample of randomly selected articles published in 2012, empirical studies were conducted in 56 different countries and corresponding authors were affiliated with 50 countries. These results suggest an ongoing trend of internationalization: Bedard et al. (2004) reported that in 2001, 44 countries provided authors for trauma related articles, compared to 18 in 1987. However, the large majority of the papers in our sample reported research in HIC. Continents such as Africa and South America were strongly underrepresented and there was a disproportionately small amount of literature on heavily populated countries such as China and India.

Our data suggest that less than 5% of recent papers on traumatic stress research result from collaborations between HIC and LMIC researchers. Moreover, 45% of the articles on LMIC studies with a HIC researcher as corresponding author did not involve any LMIC co-authors. This suggests that even when HIC researchers reach out to LMIC to study local issues, they often do not collaborate with on-site researchers on an equal basis (i.e., resulting in collaborative publications), leaving many opportunities to build capacity in LMIC untouched. Altogether, the large majority of papers in 2012 (88%) were published by research teams from HIC only.

Our results also suggest that LMIC researchers still face a significant barrier to knowledge acquisition since more than half of the publications were not available without a subscription. Since access to journal subscriptions is described as problematic in LMIC (Sharan et al., 2007), researchers in LMIC depend on open-access information. The European Journal of Psychotraumatology is currently the only domain-specific journal that is open access. Although initiatives such as Research4Life (www.research4life.org) and the International Network for the Accessibility of Scientific Publications (www.inasp.info) attempt to address the problem of high subscription fees, it is unclear to what extent they are successful in reaching out to LMIC researchers in the domain of traumatic stress (see also Olff, 2013). Our impression from conversations with LMIC researchers is that these initiatives are not known or used yet.

The present findings also indicate an imbalance in impact factors of publications, with HIC researchers publishing articles with higher impact factors than LMIC researchers. There may be several reasons for this association. Research conducted in LMIC may be of lower quality due to a lack of resources, stable research conditions and skills. It is also possible that higher impact journals have an acceptance bias toward LMIC studies and researchers (Patel & Sumathipala, 2001). The number of publications in a language other than English in our sample was similar to what Bedard et al. had reported in 2004 (5.4% compared to 6%). The dominance of the English language may mean that LMIC researchers encounter more difficulties than many HIC researchers: they compete for publication acceptance with native English speakers or if they publish in another language, do not attract a wide readership.

### Implications for global capacity building

Successful capacity building is a process of “empowering individuals, institutions, organizations and nations” (Lansang & Dennis, 2004, p. 764). On the individual level, LMIC researchers should be offered adequate training by quality training programs, distance learning options, international fellowships and collaborative memberships in research teams. HIC researchers should actively involve LMIC researchers, in particular when conducting research in their country. LMIC researchers on the other hand should be as pro-active and assertive as possible in building collaborations. Both sides can benefit from such collaboration as HIC researchers can offer their expertise in methodology and publishing internationally whilst LMIC scientists are experts on their own culture and can offer unique insights and potential explanations for findings (while we know that some HIC research teams do provide capacity building in clinical skills, our view is that this should extend to research as well).

On the level of institutions, access to recent scientific literature is a priority (Chan, Kirsop, & Arunachalam, 2005). In addition, Pang, Lansang, and Haines (2002) note in their work on “brain drain,” that the emigration of medical professionals is often due to bad working conditions, lack of funding, limited career structures and poor intellectual stimulation; factors that could be met by more international collaborations, not only of individuals but also of institutions.

On the organizational level, Schnyder's (2013) suggestions for societies of traumatic stress studies (e.g., ISTSS, ESTSS) are of value. He states that the globalization of traumatic stress research should be supported by providing more opportunity for international exchange by no cost memberships for LMIC researchers, regular meetings outside of the United States and active initiatives for international collaborations. A successful, low-cost example of the latter is the Paper in a Day event for early career researchers that led to this article.

Finally, on the level of nations, political priorities to stimulate research and education are essential. While this is often beyond the influence of individual researchers, institutions, or organizations, researchers can: (1) contribute to informing politicians of key documents on the burden of mental health problems by high-profile bodies such as the WHO, and (2) lobby for explicit inclusion of LMIC-HIC collaborations in grant systems, both in LMIC and in HIC. Several international funds such as the Wellcome Trust may function as examples in this respect.

### Strengths, limitations and conclusions

Bibliometric analyses specifically focusing on traumatic stress research previously addressed variables such as corresponding author affiliation and language of publication (Bedard et al., 2004; Figueira et al., 2007, Olff & Vermetten, 2013). However, to the best of our knowledge, this is the first analysis that additionally investigates the countries in which empirical studies have been conducted, to what extent these studies represent international collaborations, their funding and how accessible the recent literature is. Nevertheless, our analysis has its limitations. Although it is a randomly selected sample of 1,000 articles, it might not accurately reflect all research related to traumatic stress. Even though they include all languages, the databases we used appear to be primarily designed for English speaking users and therefore may not fully cover the trends in international research. In addition, our analysis was cross-sectional. Although we used the earlier reports by Bedard, Figueira, and Olff et al. (2004, 2007, and 2013, respectively) as a frame of reference, these did not capture exactly the same variables. It would be of value to conduct follow-up studies at regular intervals to track the developments in the field and also to explore more characteristics of trauma literature (e.g., whether specific types of papers are underrepresented). Finally, although our analyses indicated important gaps with regard to HIC-LMIC collaborations, in particular on LMIC studies, the present data do not offer in-depth explanations for these findings. Interviews and questionnaires may provide important insights into barriers and opportunities in this respect.

Keeping the limitations in mind, we conclude that even though there is an increasingly international perspective in traumatic stress research and an increase in research carried out by LMIC researchers, HIC research and HIC researchers still dominate the field. Our findings and reflections will hopefully stimulate initiatives to render traumatic stress research truly global.
